# HostPhinder: A Phage Host Prediction Tool

**DOI:** 10.3390/v8050116

**Published:** 2016-05-04

**Authors:** Julia Villarroel, Kortine Annina Kleinheinz, Vanessa Isabell Jurtz, Henrike Zschach, Ole Lund, Morten Nielsen, Mette Voldby Larsen

**Affiliations:** 1 Center for Biological Sequence Analysis, Department of Systems Biology, Technical University of Denmark, 2800 Kgs. Lyngby, Denmark; kortinekleinheinz@gmx.de (K.A.K.); vanessa@cbs.dtu.dk (V.I.J.); henrike@cbs.dtu.dk (H.Z.); lund@cbs.dtu.dk (O.L.); mniel@cbs.dtu.dk (M.N.); 2 Instituto de Investigaciones Biotecnológicas, Universidad de San Martín, CP(1650) San Martín, Prov. de Buenos Aires, Argentina

**Keywords:** “host specificity”, prediction, genome, k-mers

## Abstract

The current dramatic increase of antibiotic resistant bacteria has revitalised the interest in bacteriophages as alternative antibacterial treatment. Meanwhile, the development of bioinformatics methods for analysing genomic data places high-throughput approaches for phage characterization within reach. Here, we present HostPhinder, a tool aimed at predicting the bacterial host of phages by examining the phage genome sequence. Using a reference database of 2196 phages with known hosts, HostPhinder predicts the host species of a query phage as the host of the most genomically similar reference phages. As a measure of genomic similarity the number of co-occurring k-mers (DNA sequences of length k) is used. Using an independent evaluation set, HostPhinder was able to correctly predict host genus and species for 81% and 74% of the phages respectively, giving predictions for more phages than BLAST and significantly outperforming BLAST on phages for which both had predictions. HostPhinder predictions on phage draft genomes from the INTESTI phage cocktail corresponded well with the advertised targets of the cocktail. Our study indicates that for most phages genomic similarity correlates well with related bacterial hosts. HostPhinder is available as an interactive web service [1] and as a stand alone download from the Docker registry [2].

## 1. Introduction

In 2012, the World Health Organization (WHO) announced the beginning of the end of the antibiotic era, and the possible return to a time when even trivial bacterial infections could turn out to be fatal [3]. Since then, the problem of antimicrobial resistance has continued to grow and in the foreword to the WHO report “Antimicrobial resistance: global report on surveillance 2014” it is stated that “A post-antibiotic era-in which common infections and minor injuries can kill-far from being an apocalyptic fantasy, is instead a very real possibility for the 21st century” [4]. As emphasized by WHO there is an urgent need for treatment alternatives, one such being bacteriophages (phages). The idea of using phages for the treatment of bacterial infections dates back to 1919, when French-Canadian microbiologist Félix d’Herelle used them for treating a patient with severe bacillary dysentery [5]. For a number of historical reasons, phage therapy never became general practice in the West, although it has been used extensively in countries from the former Eastern bloc [6,7,8,9]. Several recent studies from the West have also demonstrated the effectiveness of phages as antibacterial treatment [10,11,12,13], and more countries are currently revisiting phage therapy [14,15]. Phages have furthermore been suggested for use in the agriculture and food industries [16,17]. Examples include their use for reducing *Campylobacter jejuni* colonisation of broiler chickens [18] and the growth of *E. coli* in milk [19].

For a phage to successfully infect a bacterial host, the phage must adsorb to the bacterial surface through recognition of specific host receptors, e.g., proteins, LPS, or cell wall polysaccharides. Phage adsorption to an appropriate surface receptor is, however, only the first step required for successful infection. Several host defence mechanisms must also be overcome: Restriction-Modification (RM) systems have been shown to be present in more than 90% of sequenced bacterial genomes [20]. These systems include restriction enzymes that degrade incoming phage DNA with appropriate target sequences. Some bacteria contain Clustered Regular Interspaced Short Palindromic Repeats (CRISPR) loci, which together with the CRISPR-associated (cas) genes encode an adaptive anti-phage immune system [21]. Phage abortive systems (Abi systems) allow infected bacteria to commit “altruistic suicide” thereby preventing the spread of the phage within the bacterial community [22]. Other factors such as successful gene transcription and translation based on amino acid or tRNA availability further limit the host range [23]. Bacteria and phages have from the outset of their coexistence been engaged in a vehement arms race leading to intricate coevolutionary processes, and for each of the defence mechanisms mentioned above, examples exist of phages that have evolved to circumvent them [24,25]. The arms race has contributed to bacterial as well as phage diversity [26] and entails that phage host determination is influenced by multiple genes and genome features distributed across the phage genome. Although examples exist of phages that have extended their host range based on only a few mutations [27], the extended host range is typically limited to different strains of the same species. Apart from polyvalent enterobacteria phages, which are able to infect members of phylogenetically linked genera within the *Enterobacteriaceae* family, e.g., *Escherichia*, *Shigella*, and *Klebsiella* [28,29], most phages have been found to be specific to a particular genus [30]. This has been indicated by studies examining proteins, not entire proteomes [31], as has the “Phage Proteomic Tree”, which is based on completely sequenced phage genomes [32], and analysis of genome type for Mycobacteriophages and host preference [33].

In this study, we extend the observation that genetically similar phages often share the same bacterial host species and hypothesize that it should be possible to predict the host species of a phage by searching for the most genetically similar phages in a database of reference phages with known hosts. In the developed method, called HostPhinder, genetic similarity is defined as the number of co-occurring k-mers between the query phage and phages in the reference database. K-mers are stretches of DNA with a length of k, and their use as a measure of genetic relatedness dates back to Woese and Fox and their groundbreaking paper from 1977, which uncovered Archaea as a separate branch in the tree of life [34]. Woese and Fox limited their analysis to k-mers (they used the term oligonucleotides) in 16S (18S) ribosomal RNA, but since phages do not have 16S rRNA genes or any other genes which are common to all phages [32], and because high-throughput sequencing methods have made the entire genome of phages easily available, HostPhinder examines the complete genome. Further, for bacteria we have previously shown that the co-occurrence of k-mers across the entire genome performs superior to other whole-genome or single locus based approaches for inferring genetic relatedness [35]. The splitting of entire phage genomes into overlapping k-mers may furthermore be an advantage in relation to the highly mosaic phage genome structure [36,37].

We believe that a method enabling prediction of the bacterial hosts of phages will be useful for several reasons. Firstly, phages have for many years been used to treat bacterial infections in countries belonging to the former Eastern bloc. The Eliava Institute in Tbilisi, Georgia has in particular been dominant in this regard and produce cocktails containing a mixture of phages for a range of bacterial infections. One of the steps towards adopting phage therapy in the West, is likely to be a full characterization of the content of these cocktails, which due to the way they are manufactured is not known [38]. Further, the current approach to exploration of many ecological niches is done by untargeted sequencing of samples isolated directly from the environment, so called metagenomics. This enables identification of phage and bacterial sequences without knowledge of the link between them, and importantly also enables identification of bacteria, and hence phages, that cannot be cultured. HostPhinder could help establish the link between phages and bacteria, which might be an important step towards understanding, e.g., the microbiome of the human gut, and possibly associations between the microbiome and clinical parameters of the human host [39].

## 2. Materials and Methods

### 2.1. Whole Genome Phage Sequences from Public Databases

A set of public phage Whole Genome Sequences (WGS) was collected in August 2014: First, lists of phage WGS IDs were obtained from Phages.ids–VBI mirrors page [40], the NCBI viral Genome Resource [41], the EMBL EBI phage genomes list [42], and the phagesdb databases for Mycobacteriophages [43], Arthrobacter [44], Bacillus [45], and Streptomyces [46]. The resulting unique list of IDs was uploaded to the Batch Entrez service of NCBI to retrieve the corresponding WGS. Furthermore genome sequences were downloaded from the PhAnToMe genomes database and from NCBI searching for “(phage [Title]) AND complete genome”.

**Figure 1:**
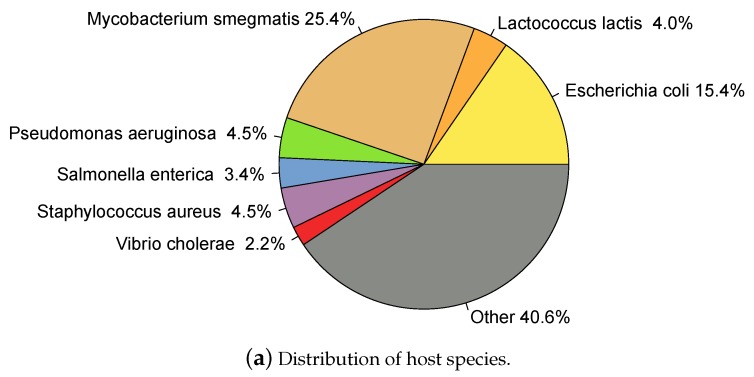

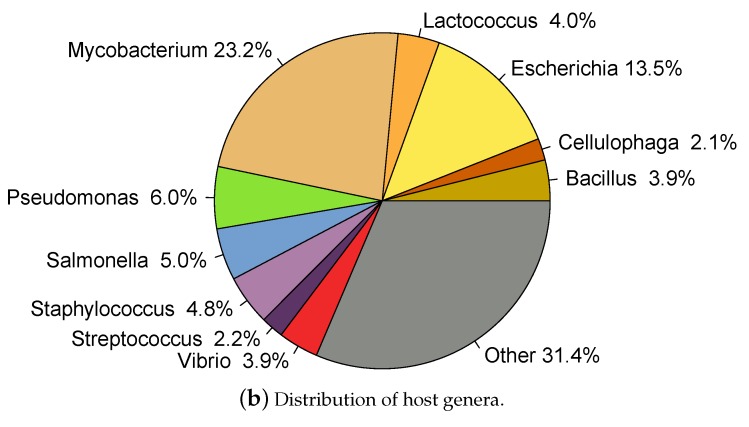
Hosts represented in the database. Species (**a**) and genera (**b**) representations are displayed in the same genera-colour code.

Only entries indicating "complete genome" in the DEFINITION field of the GeneBank file and which host taxonomy was specified at least at the genus level were included. Entries annotated as "prophage" in the DEFINITION were removed. Hosts annotated as *Salmonella Typhimurium* were re-annotated as *Salmonella enterica* according to current nomenclature [47]. Finally, only the genus was taken into account for hosts with species specified as "sp." followed by an alphanumeric code; for example *Synechococcus* sp. *WH7803* was re-annotated as *Synechococcus*. 2196 phages had annotated host genus, here called ${\mathrm{phages}}_{\mathrm{genus}}$ dataset, and of these, 1871 had annotated species as well, ${\mathrm{phages}}_{\mathrm{species}}$. A total of 209 different host species and 129 different genera were represented among the phages (this data is available in HostPhinder’s repository [48]). Figure 1 shows the distribution of hosts in the dataset.

### 2.2. Data Partitioning and Clustering

In this study, a 4-fold cross validation setup was used to assess the ability of the host prediction method to generalize to previously unseen data. Five data partitions were made, and one partition, ${\mathrm{phage}}_{\mathrm{eval}}$ was left aside during the entire process of parameter optimization. Once the parameters were optimized, the prediction accuracy was evaluated on this ${\mathrm{phage}}_{\mathrm{eval}}$ set, using the entire ${\mathrm{phage}}_{\mathrm{train},\mathrm{test}}$ set as reference database (Supplementary Materials Figure S1). In this setup, the performance of the evaluation set is hence completely unbiased towards the model parameter optimizations.

A reliable, *i.e.*, not overfitted, evaluation can only be made if phage genomes in the training-test and evaluation sets are not too similar to each other. Indeed, if a phage genome in the training set is almost identical to a genome in the evaluation set, it would be a simple task for HostPhinder to predict its host, leading to an overestimation of the method’s ability to generalize to previously unseen data. To avoid such a bias we clustered the genomes according to 16-mer similarity by means of a Hobohm 1 approach [49]. The Hobohm approach consists in the formation of a final list of representative phage genomes, here called seeds. After the first sequence in a randomly sorted list enters the seed list and forms a seed, the following sequences are each checked for similarity (number of overlapping 16-mers) to each seed in the final list. Only if significantly different to the seed sequences, the new sequence will be included in the seed list. Otherwise, it will be linked to the most similar seed as member of the same cluster. The similarity between two genomes was measured in terms of ${\mathrm{frac}}_{q}$ (see Equation (4) in section “K-mer-based resemblance measures”) using a threshold ${\mathrm{frac}}_{q}$ > 0.7. This threshold was chosen because the resulting clustering was most similar (93%) to the clustering obtained with a BLAST-Hobohm1 approach, where the similarity threshold was set to >90% genomewide ID (data not shown). The k-mer-Hobohm1 analysis resulted in 293 clusters with at least 2 sequences and 1121 singlets. The total number of seeds was hence 1414 containing 1 to 97 sequences. To separate the clustered phages in train-test and evaluation sets, the 1414 seeds were sorted by host alphabetical order, and secondly by size and alternately distributed between 5 partitions. This assured an equal host and genome size representation among partitions. Finally remaining members of each cluster were integrated into the partition of their respective seed. Sequences within the same cluster shared the host; therefore the unbiased host distribution was maintained also after integrating members of the clusters in each partition (see Supplementary Materials Figure S2). Subsets of each of these partitions were made, which comprised all phages that contained information about the species of the host, overall constituting the ${\mathrm{phages}}_{\mathrm{species}}$ dataset. The host and size distribution between partitions remained conserved (see Supplementary Materials Figures S2–S4). As stated above, one partition was next left aside for final evaluation, ${\mathrm{phages}}_{\mathrm{eval}}$, and the remaining 4 formed the train-test set, ${\mathrm{phages}}_{\mathrm{train-test}}$. The final ${\mathrm{phages}}_{\mathrm{train-test},\mathrm{genus}}$ set contained 1818 phages (115 genera and 190 species), the ${\mathrm{phages}}_{\mathrm{eval},\mathrm{genus}}$ set contained 378 phages (72 genera, 96 species), while the ${\mathrm{phages}}_{\text{train-test,species}}$ set consisted of 1546 phages and the ${\mathrm{phages}}_{\mathrm{eval},\mathrm{species}}$ set consisted of 325 phages (data available in HostPhinder’s repository [48]).

### 2.3. K-mer-Based Resemblance Measures

Under the assumption that phages infecting the same bacterial host share genomic features, the host of a query phage should be predictable by searching for the most genomically similar phages in a reference database of phages with annotated hosts. The reference database was build from phage genome sequences and their reverse complements by splitting both into k-mers and sliding a window of length k along the sequences with step-size 1.

Query sequences were likewise split into k-mers, and for each reference sequence *i* having at least one k-mer in common with the query, a score, $S_{i}$, was defined as the number of identical unique k-mers between query and template. This score was subsequently used to determine the expectation value $E_{i}$:

(1) $$E_{i}=N_{\mathrm{Hits}}\frac{l_{u,i}}{L_{u,\mathrm{tot}}}$$

where $N_{\mathrm{Hits}}$ is the sum of scores over all references, $l_{u,i}$, is the total number of unique k-mers found in the reference sequence *i* and in its reverse complement and $L_{u,\mathrm{tot}}$ is the sum of unique k-mers over all references in the database. This expectation value was used to obtain a z-score:

(2) $$z_{i}=\frac{S_{i}-E_{i}}{\sqrt{S_{i}+E_{i}+\eta }}$$

with η=0.001 being a pseudocount used to avoid division by zero. Using SciPy, a two-sided *p*-value was generated from the z-score. All *p*-values were corrected using the Bonferroni method [50] by multiplying each *p*-value by the number of reference phages in the database:

(3) $$p_{\mathrm{corr}}=p_{i}*N_{\mathrm{ref}}$$

where $N_{\mathrm{ref}}$ is the number of reference sequences in the database. HostPhinder outputs only significant hits, *i.e.*, $p_{\mathrm{corr}}<0.05$. Additionally, the values ${\mathrm{frac}}_{q,i}$ and ${\mathrm{frac}}_{d,i}$ were estimated. They represent the ratio of the score and the number of unique k-mers in query and reference sequences respectively:

(4) $${\mathrm{frac}}_{q,i}=\frac{S_{i}}{q_{u,i}+\eta }$$

where $q_{u,i}$ is the number of unique query k-mers and η=0.001 avoids division by zero. The value of ${\mathrm{frac}}_{q,i}$, falling between 0 and 1, gives a direct indication of how much of the query sequence matched to the reference phage.

(5) $${\mathrm{frac}}_{d,i}=\frac{S_{i}}{l_{u,i}+\eta }$$

where $l_{u,i}$ is the number of unique k-mers in the reference sequence and in its complement. Therefore, ${\mathrm{frac}}_{d,i}$ falls between 0.5 and 1 if query and reference are identical, depending on the number of additional unique k-mers found in the reversed complement. The two measures are hence not directly comparable. Finally the coverage was determined as a measure of how much of the reference sequence is covered by the total number of k-mers in the query that match the reference:

(6) $${\mathrm{coverage}}_{i}=\frac{2q_{\mathrm{matched},i}}{l_{u,i}+\eta }$$

where $q_{\mathrm{matched},i}$ is the total number of k-mers in the query that were matched to reference *i*, and $l_{i}$ is the total number of k-mers in the reference. Both of these values include identical k-mers and do not only count unique k-mers. The factor 2 is included to account for the additionally used reverse complement sequence of the reference to obtain $l_{i}$. The coverage can be larger than 1 if the query contains k-mers that could be matched multiple times.

### 2.4. Determining the Measure and Selection Criteria for Final Prediction

As described above, 5 measures were calculated for the similarity of a query phage to each of the phages in the reference database: score, z-score, ${\mathrm{frac}}_{q}$, ${\mathrm{frac}}_{d}$, and coverage. The optimal measure was determined in a simple 4 fold cross-validation setup. Here in turn, 3 of the 4 data sets were used as reference database for predicting the host for each query phage in the left out test set (see Supplementary Materials Figure S1, left). The host was inferred from the host of the reference phage with the highest value of similarity measure. This was repeated 4 times so that all 4 partitions were used as test set, and an overall performance for the given measure was calculated by concatenating the predictions of the 4 test sets. For each measure the average and interval of confidence was assessed through 100 bootstrap resamplings with replacement for each test set and calculating the overall accuracy. On a pairwise comparison based on 1000 bootstrap resamplings, coverage outperformed the other measures and was therefore chosen for further analysis. A number of different selection criteria can be used for the final prediction of the host of a query phage. We tested and compared the efficacy of 4 selection criteria that are each described in detail below.

#### 2.4.1. Criterion 1: Host of Best-Matching Reference Phage

The host of the reference phage with the highest coverage value was selected as predicted host. This is the selection criterion used above to define the optimal similarity measure.

#### 2.4.2. Criterion 2: Majority Host among Top-10 Reference Phages

As predicted host, the most abundant host among the hosts of the top 10 reference phages with the highest coverage values was selected. In case of a tie, the most abundant host with the highest coverage, was selected.

In cases where the coverage of non-top reference phages is far below the coverage of the top reference phage, it might not be advantageous to consider them in the selection criterion. To accommodate this, two additional criteria, criteria 3 and 4, were developed.

#### 2.4.3. Criterion 3: Majority Host among Reference Phages above Coverage Threshold

**Figure 2:**
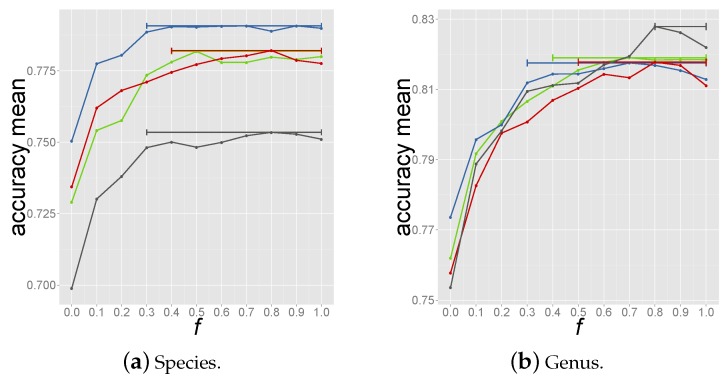
Accuracy *vs.* *f* values obtained from the 4 loops of inner cross validation. Each dot represents the averaged accuracy for species (a) and genus (b) prediction over 100 bootstrap resamplings. The bars cover the range of *f* values for which the accuracy is 99% the highest accuracy in the specific tripartite set.

As predicted host, the most abundant host among the phages with a coverage value above a given threshold was selected. The threshold was defined as a fraction of the highest coverage:

(7) $${\mathrm{coverage}}_{\mathrm{threshold}}=f{\mathrm{coverage}}_{1}$$

where *f* (fraction) is a number in the range 0.0–1.0. Note that f=0.0 means considering all significant predictions, whilst f=1.0 corresponds to selecting the host of the reference phage with the highest coverage (criterion 1). The optimal value of *f* was determined through a nested 3 fold cross-validation to avoid biased estimates of performances that would result from using the same cross validation used to select the optimal criterion. Here in turn, 3 data partitions were used as tripartite train-test set in a procedure called inner cross-validation. Within the tripartite set, 2 partitions were sequentially used as reference database for predicting the host for the left out test set using Equation (7) for a given value of *f*. This was repeated 3 times within each tripartite set so that all 3 partitions were used as test set and an overall performance for the given *f* value was calculated (see Supplementary Materials Figure S5). For each *f* value the average accuracy was assessed through 100 bootstrap resamplings with replacement for each inner cross validation loop. The same procedure was repeated 4 times so that each tripartite combination was analysed leading to 4 estimates of the optimal *f* value. The accuracy *vs.* *f* values curves are shown in Figure 2 for prediction of species and genus. The horizontal bars span *f* values that yield at least 99% of the highest accuracy in the relative tripartite combination. Given these performance curves, an *f* value of 0.8 was chosen within the highest performance range, Figure 2.

#### 2.4.4. Criterion 4: Summing up Normalized Coverage Values of Phages with Same Host

In the scoring method, coverage values of all significant reference phages were normalised by division by the highest coverage, ${\mathrm{coverage}}_{1}$, and raised to the power of an arbitrary number, α>0.

(8) $${\mathrm{score}}_{i}={\left(\frac{{\mathrm{coverage}}_{i}}{{\mathrm{coverage}}_{1}}\right)}^{\alpha }$$

**Figure 3:**
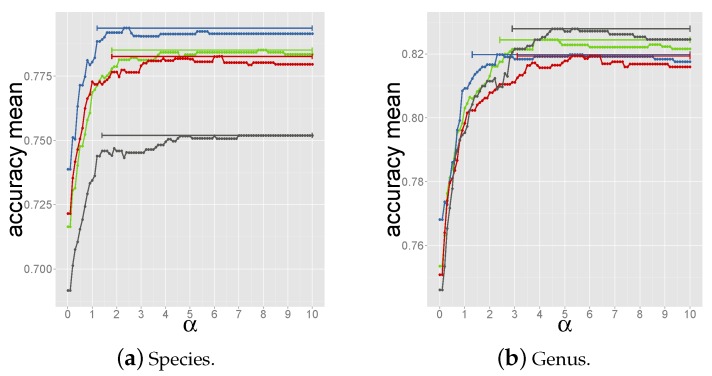
Accuracy *vs.* *α* values for prediction of species (a) and genus (b) in each tripartite set. Each dot represents the averaged accuracy over 100 bootstrap resamplings. The bars cover the range of *α* values for which the accuracy is 99% the highest accuracy.

Next, scores of hits with the same host were summed up and the host was predicted as the one with the highest score. The higher the value of *α*, the higher the score of the first hit, the closer this method is to criterion 1. Values of *α* in the range 0.0–10.0 were tested. As for the criterion 3, the optimal *α* was determined through a nested 3 fold cross-validation setup (see Supplementary Materials Figure S5) and led to the selection of α=6.0 within the range that yielded the highest accuracy in the 4 tripartite train-test sets (see Figure 3).

### 2.5. Programming Language and Speed of Execution

The algorithm was written in Python and Bash.

On an Intel(R) Xeon(R) CPU E5-4610 v2 @ 2.30GHz computer, using 2 cores and 10 GB RAM, HostPhinder average running time is of 61.1662 s for host species prediction and 109.622 s for genus prediction. The longer runtime for genus prediction is due the larger database used for genus predictions. These values were calculated on the evaluation set.

### 2.6. BLAST Evaluation

The accuracy of the HostPhinder k-mer based approach was compared to the state-of-the-art tool in bioinformatics, BLAST [51]. BLAST performance was assessed on the ${\mathrm{phages}}_{\mathrm{eval}}$ set using the ${\mathrm{phages}}_{\text{train-test}}$ set to create a local nucleotide BLAST database. The host associated to the hit with the lowest E-value and secondarily highest bit score was returned as prediction.

### 2.7. Establishing an Evaluation Set of Predicted Prophages

The PhiSpy prophage prediction tool [52] was used to predict prophages in 2679 complete bacterial genomes collected from NCBI [53]. PhiSpy was run once on each genome resulting in a total of 7559 predicted bacterial prophages in 2074 genomes. Of these, 2796 were from bacterial species that were also included in the HostPhinder reference database. In the following, these predicted prophages will be referred to as the ${\mathrm{prophages}}_{\mathrm{species}}$ set. A total of 4639 predicted prophages were from genera that were included in the reference database of HostPhinder. They will be referred to as the ${\mathrm{prophages}}_{\mathrm{genus}}$ set.

Furthermore 261 manually verified prophages were downloaded from PhiSpy and phage_finder directories from Phantome [54] and HostPhinder prediction was tested on them.

### 2.8. Host Prediction of INTESTI Bacteriophage Cocktail

The Georgian George Eliava Institute of Bacteriophages, Microbiology and Virology has developed phage cocktails (mixtures of phages) since the 1950s. One of these, the INTESTI bacteriophage cocktail, claims to contain sterile filtrates of phage lysates effective against *Staphylococcus*, *Enterococcus*, *Proteus*, *Shigella*, *Salmonella*, *Escherichia coli*, and *Pseudomonas aeruginosa* for the treatment of intestinal bacterial infections. The cocktail was sequenced directly on an Illumina MiSeq platform and de novo assembled to contigs, which were further grouped into 19 draft genomes each hypothesized to represent close to complete phage genomes, and 4 smaller groups hypothesized to represent fragments of phage genomes previously described [38]. The host genus and species of each of these 23 groups was predicted by the final HostPhinder method using the 4th criterion with α=6.0.

## 3. Results

In this study, we developed and benchmarked HostPhinder, a bioinformatics tool for predicting the bacterial host species of phages. The method is based on the assumption that genetically similar phages are likely to share bacterial hosts. For performing the predictions, HostPhinder relies on a reference database in which WGS data from phages with annotated hosts have been split into k-mers. The genomes of the query phages for which the hosts should be predicted are likewise split into k-mers, and the number of co-occurring k-mers between the query phage and the phages in the reference database is used as a measure of genetic similarity.

### 3.1. Developing and Benchmarking the HostPhinder Method

Initial analysis on a small dataset indicated that k-mers of length 15–20 nt led to comparable predictive performances. In contrast, shorter k-mers were too unspecific and led to a lower final accuracy, while longer k-mers were too specific and led to more query phages for which no predictions at all could be made (data not shown). Based on these results and a previous study that showed 16-mers to be optimal, when using a k-mer based approach for bacterial species identification [35], 16 was chosen as the k-mer length in the following.

In the initial testing of the basic genetic similarity assumption of HostPhinder, 5 measures were evaluated for estimating the similarity of the query phage to the reference phages as described in Materials and Methods. For each measure, the query host was inferred from the host of the reference hit with the highest similarity. Table 1 shows the performance of each similarity measure in this initial testing.

The measures’ accuracies in predicting the query phage host species of the training-test set were pairwise compared by 1000 bootstrap resamplings with replacement. Coverage performed significantly better than other measures (*p*-value < 0.05), apart from ${\mathrm{frac}}_{d}$, which in turn did not significantly outperformed coverage. Since coverage showed the highest performance in predicting the host species, it was chosen as the measure used when further optimizing HostPhinder prediction at the species level. Next, the performance of 4 scoring methods for host selection was compared (see Material and Methods for criteria description and parameter optimization). For each selection criterion only significant hits were considered ($p_{\mathrm{corr}}<0.05$) and the number of queries with predictions was constant for all criteria allowing a direct comparison of criteria efficacy. Using the model parameters determined above, the 4 criteria were compared in terms of overall accuracy in a 4 fold cross-validation system. In turn, 3 of the 4 partitions were used as reference database for predicting the host for the left out test set using each criterion. This was repeated 4 times so that all 4 partitions were used as test set, and an overall performance for the given criterion was calculated. For each criterion the average and interval of confidence was assessed through 100 bootstrap resamplings with replacement for each test set and calculating the overall accuracy. Table 2 shows the overall accuracy on ${\mathrm{phages}}_{\text{train-test,genus}}$ and ${\mathrm{phages}}_{\text{train-test,species}}$ sets for each criterion on genus and species level, respectively. Bacterial host genera and species were not predicted for 5.8% ${\mathrm{phages}}_{\mathrm{train-test},\mathrm{genus}}$ and 5.6% ${\mathrm{phages}}_{\mathrm{train-test},\mathrm{species}}$ phages respectively.

Criterion 4 with α=6.0 had the highest predictive value, with an accuracy of 79% and 84% for species and genus respectively, even though it only significantly outperforms criterion 2.

Some hosts are substantially more frequent than others in the data set. This could potentially lead to a bias in the prediction, and a subsequent sub-optimal predictive performance. To investigate this, modified versions of criteria 2–4 were tested, where the sequences in the reference database were clustered according to Hobohm 1 algorithm [49], and only the highest scoring element within one cluster was used in the prediction schema. This did not, however, improve the performance.

Based on the above benchmarking procedures, the final method called HostPhinder was developed. The reference database was generated by splitting all phage genomes in the entire phage set into 16-mers using a step-size of 1. After searching through the database, HostPhinder examines the coverage measure and creates a hits list, *i.e.*, phages significantly similar to the query. The final host species and genus is given according to criterion 4 with an α=6.0. HostPhinder is freely available as a web server [1] and as a Docker image [2].

### 3.2. Evaluating HostPhinder’s Performance on Complete and Partial Genomes

**Figure 4:**
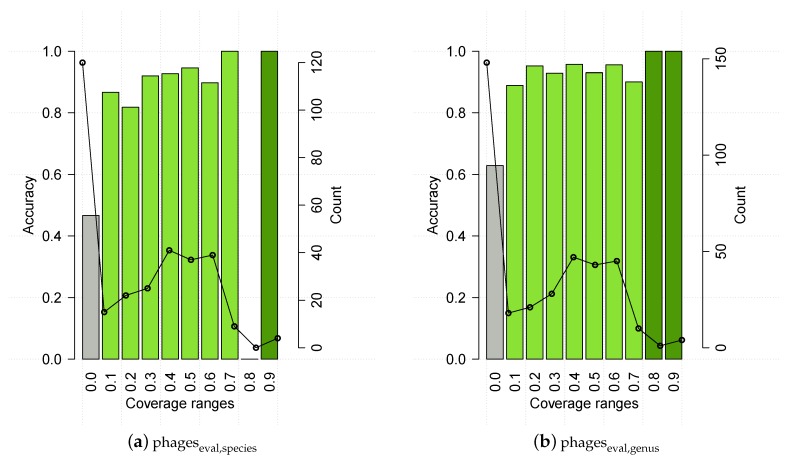
HostPhinder’s accuracy (bar) and prediction counts (line) on ${\mathrm{phages}}_{\mathrm{eval}}$ at different coverage ranges. The values displayed on the *x* axis are the lower limit of that range. With exception of the last bin which includes all entries with coverage >0.9, all ranges are right-closed with upper limit *x* + 0.1. Poorly reliable results are in grey, while reliable and highly reliable results are in green and dark green respectively. Results on HostPhinder’s web server [1] are displayed using the same colour code.

HostPhinder was evaluated on the ${\mathrm{phages}}_{\mathrm{eval},\mathrm{genus}}$ and ${\mathrm{phages}}_{\mathrm{eval},\mathrm{species}}$ sets containing phages from public databases. HostPhinder was able to correctly predict the bacterial host species and genera of 74.24% ± 0.270% and 81.39% ± 0.206% of the phages respectively. In the evaluation set, 4.0% (3.44%) of the phages could not be matched to any phage in the database when predicting on species (genus) level. We speculated that the accuracy of the HostPhinder method is depending on the coverage value of its prediction. That is, the higher the coverage value, the higher the accuracy. To quantify if this is indeed the case, we show in Figure 4 the accuracy on the evaluation set at different intervals of the coverage value. No hit appeared to have range 0.8 < coverage ≤ 0.9 for species. For species as well as genus level, it can be seen that predictions based on a coverage value below 0.1 are only correct for 47% (species) and 63% (genus) of the phages. At the other end of the scale, predictions based on a coverage value above 0.7 (species) and 0.8 (genus) are correct in all instances.

**Figure 5:**
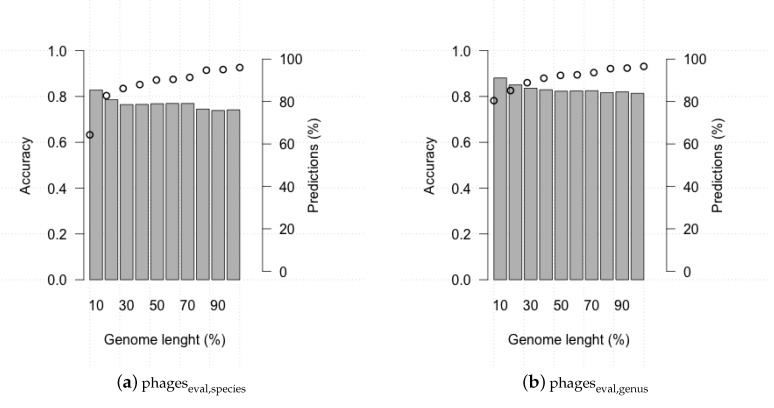
HostPhinder’s accuracy (bar) and percentages of predictions (dots) on ${\mathrm{phages}}_{\mathrm{eval}}$ at different percentages of genome length from 10% to 100% of total genome length.

Assembly of metagenomic samples often do not results in entire phage genomes. To assess how the completeness of a phage genome affects HostPhinder performance, we ran the tool on the evaluation set where each genome was gradually reduced by 10%, 20%, ... ,90% of its total length. Figure 5 shows the accuracy and the number of predictions for each percentage of genome length. HostPhinder maintained the prediction accuracy but made gradually fewer predictions as the fraction of genome given as query is decreased.

Generally, HostPhinder returned predictions at 10% genome length for those genomes which prediction at complete genome length had a higher coverage. The average coverage for predictions made at complete genome length but not at 10% genome length was 0.023, while the average coverage for commonly predicted was 0.36.

We next examined if HostPhinder always correctly predicted particular host species or genera (Table 3). Only hosts occurring at least 3 times in the ${\mathrm{phages}}_{\mathrm{eval}}$ set are listed. All phages in the ${\mathrm{phages}}_{\mathrm{eval}}$ set that target these hosts listed in Table 3 were correctly predicted. Additionally, none of these hosts were erroneously predicted as targets of other phages.

HostPhinder also worked effectively for predicting the host of phages, which according to the initial clustering were of different types; in fact in the HostPhinder dataset there are 14 different types of *Enterococcus faecalis* phages, 13 types of *Listeria monocytogenes* phages and 21 types of *Vibrio cholerae* phages and all phages known to infect these host have been correctly predicted, see Table 3.

**Figure 6:**
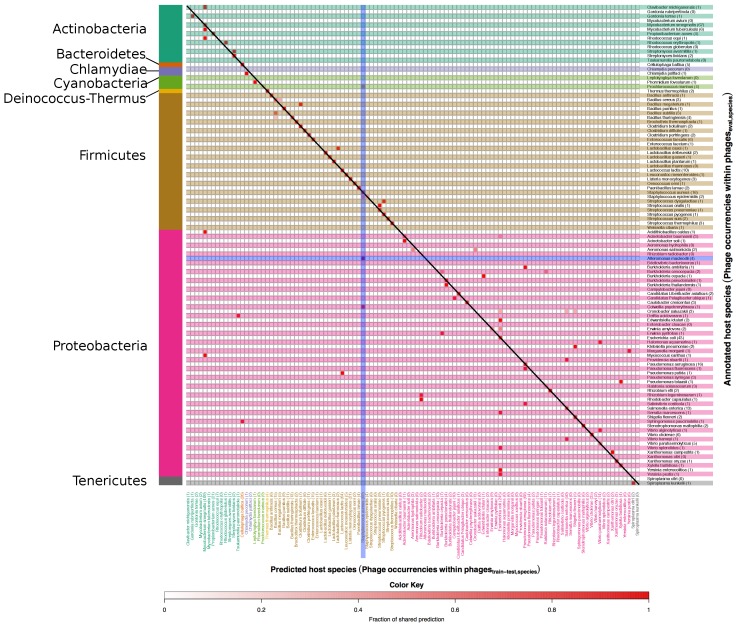
Heatmap of annotated *vs.* predicted host species in the ${\mathrm{phages}}_{\mathrm{eval},\mathrm{species}}$ set. In this figure correct as well as mispredicted host species can be seen. Annotated host species are listed along the *y* axis, while predicted ones are on the *x* axis. The number after each species on the *y* axis and the *x* axis also indicate the occurrences of phages in the ${\mathrm{phages}}_{\mathrm{eval},\mathrm{species}}$ and in the ${\mathrm{phages}}_{\mathrm{train-test}}$ respectively. Host species are grouped according to the respective phylum, which are indicated on the left side of the figure. The colour scale indicates the fraction of phages predicted as targeting a particular host and goes from white, no phages, to red, 100% of the phages. Accordingly, the colour itself is not an indicator of correctness of the prediction, and red colours along the diagonal represent correct predictions.

**Figure 7:**
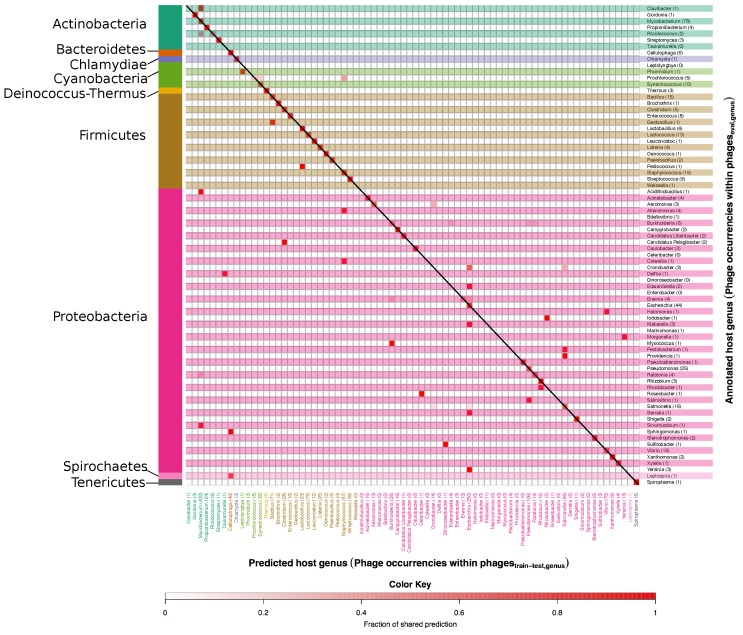
Heatmap of annotated *vs.* predicted host genera in the ${\mathrm{phages}}_{\mathrm{eval},\mathrm{genera}}$ set. In this figure correct as well as mispredicted host genera can be seen. Annotated host genera are listed along the *y* axis, while predicted ones are on the *x* axis. The number after each genus on the *y* axis and the *x* axis indicate the number of occurrences of phages in the ${\mathrm{phages}}_{\mathrm{eval},\mathrm{genus}}$ and ${\mathrm{phages}}_{\mathrm{train-test,genus}}$ respectively. Host genera are grouped according to the respective phylum, which are indicated on the left side of the figure. The colour scale indicates the fraction of phages predicted as targeting a particular host and goes from white, no phages, to intense red, 100% of the phages. Accordingly, the colour is in itself not an indicator of correctness of the prediction, and red colours along the diagonal represent correct predictions.

Figure 6 and Figure 7 show right and wrong predictions for species and genera respectively. To ease comprehension of the plots, hosts were grouped by phyla, which are displayed on the left side of the figures. Rows are alternatively shaded and column names are enhanced with the same colour of the phylum of belonging. The heatmaps are read from right to left and then downwards; expressely, the phage related to the host identified by the row name, on the right, was predicted (red intensity of the cell) to infect the host identified by the column name in the lower part of the figure. As an example, *Alteromonas macleodii* phages, the row encompassed in a blue horizontal box in Figure 6, occurred four times in the ${\mathrm{phages}}_{\mathrm{eval},\mathrm{species}}$ set, as indicated by the number within parenthesis beside the host name, and all of them were wrongly predicted to be *S. aureus* phages (vertical blue box) as indicated by the intense red colour of the square in the intersection between the two blue boxes; of note, there were 69 *S. aureus* phages in the ${\mathrm{phages}}_{\mathrm{train-test,species}}$ data set and no *Alteromonas macleodii* phages.

At species level, phages with mispredicted hosts are often predicted to target a host of the same genus as the annotated host (see small deviations from the diagonal in Figure 6). As examples, the 3 phages annotated to target *Bacillus subtilis* are predicted to target either *B. subtilis* or *Bacillus cereus*. For some phages the mispredicted host is, however, of an entirely different genus, e.g., the phage annotated to target *Yersinia enterocolitica* and the phage annotated to target *Yersinia pestis* are both predicted to target *E. coli*. For species as well as genera there is a tendency that phages with mispredicted hosts are predicted to target the most frequent hosts in the ${\mathrm{phages}}_{\mathrm{train-test}}$ set, e.g., *E. coli* and *Mycobacterium smegmatis* on species level and *Escherichia* and *Mycobacterium* on genus level. What is important to note is that inaccurate predictions were finding related hosts. For example, imprecise predictions of phages infecting *Proteobacteria* (the ones within the brown region) were still falling within the phylum of *Proteobacteria*. This indicates a relatedness in terms of genome sequence among phages infecting different hosts belonging to the same phylum.

### 3.3. Comparing HostPhinder to BLAST

Next, the HostPhinder performance on ${\mathrm{phages}}_{\mathrm{eval}}$ was compared to BLAST. Table 4 summarises the results.

HostPhinder was able to make host predictions for more phages than the BLAST-based method. For the phages that both methods were able to make a prediction for, HostPhinder outperformed BLAST on both genus and species level. The observed better performance of HostPhinder on species level is significant (*p* < 0.05). HostPhinder correctly predicted 25% among 24 (genera) and 10% among 20 (species) predictions not covered by BLAST. Moreover when inferring the host genus of a phage for which HostPhinder gave no prediction, BLAST match to the most closely related phage resulted in the wrong prediction.

### 3.4. HostPhinder’s Performance on Predicted Prophages and Establishment of Confidence Threshold

To further evaluate the performance of HostPhinder and to establish a confidence threshold for the predictive value, we examined if HostPhinder was able to identify the bacterial hosts of predicted prophages on the premise that prophages are phages that have at one point infected the host that they are currently found in. The predicted prophages provide a dataset diverse enough to define a reliability threshold that can be generalized and applied to previously unseen data. For this purpose, we predicted prophages in 2679 bacterial genomes using PhiSpy [52]. Without any threshold value set, HostPhinder was able to correctly predict approximately 45% and 47% of the species and genus respectively. The accuracy was calculated over the number of phages that HostPhinder was able to make a prediction for.

**Figure 8:**
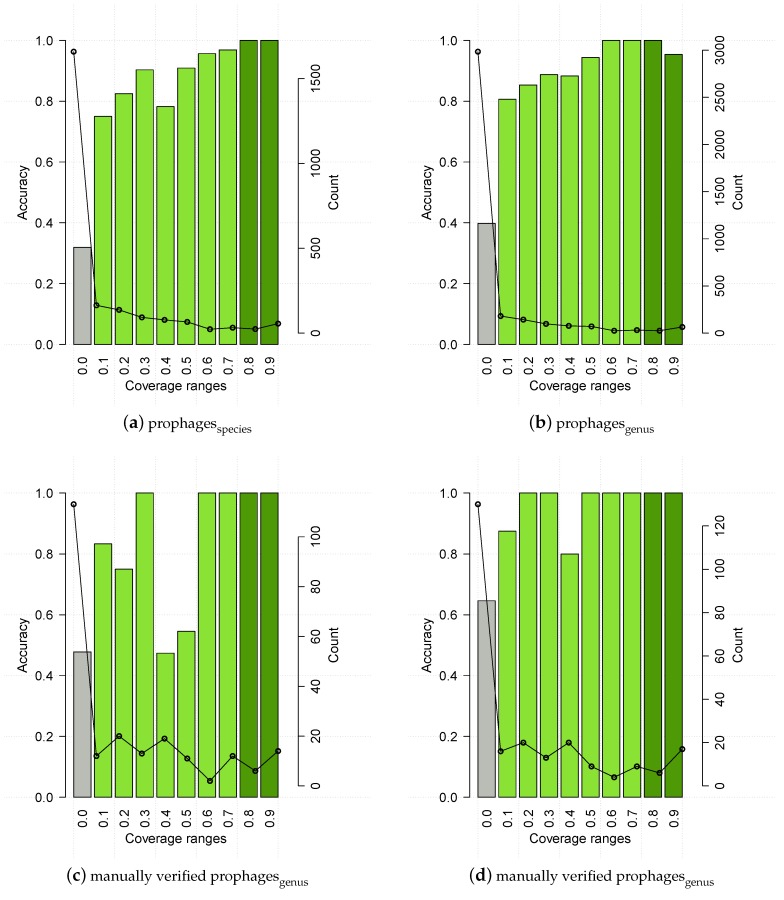
HostPhinder’s accuracy (bar) and prediction counts (line) on prophages predicted by PhySpy, upper panels, and manually verified prophages, lower panels, at different coverage ranges. The values displayed on the *x* axis are the lower limit of that range. With exception of the last bin which includes all entries with coverage >0.9, all ranges are right-closed with upper limit *x* + 0.1. Poorly reliable results are in grey, while reliable and highly reliable results are in green and dark green respectively.

As for ${\mathrm{phages}}_{\mathrm{eval}}$, the results on PhiSpy predicted prophages were binned into coverage ranges (Figure 8, upper panels). The accuracy pattern for prophages generally resembled the one for the evaluation set, *i.e.*, it had low accuracy for coverage ≤1, and 100% accuracy above a certain threshold, which in this case is 0.8 for species. There is an unexpected drop in accuracy for coverage values >0.9 (genus), which a bootstrap analysis proved non significant (*p* > 0.05). To further cofirm the thresholds, we ran HostPhinder on 261 manually verified prophages, downloaded from PhAnToMe.org, which resulted in 63.57 % ± 0.356 % and 78.69 % ± 0.262 % prediction accuracy of species and genus respectively. Accuracy distribution for this dataset among different coverage ranges can be seen in Figure 8, lower panels. Based on observations ${\mathrm{phages}}_{\mathrm{eval}}$ and on prophages, HostPhinder considers trustable results with coverage value higher than 0.1, and it applies a conservative threshold of 0.8 to distinguish highly trustable results.

### 3.5. Host Analysis of Phages from Therapeutic Phage Cocktail from the Georgian George Eliava Institute

In a recent study, we examined the content of an INTESTI bacteriophage cocktail from the Georgian George Eliava Institute. According to the packing, the cocktail is effective against *Staphylococcus*, *Enterococcus*, *Proteus*, *Shigella*, *Salmonella*, *Escherichia coli*, and *Pseudomonas aeruginosa* infections [38]. A total of 19 phage draft genomes were identified that were hypothesized to represent close to complete phage genomes. An additional set of four sequences represented fragments of phage genomes. Here, we used HostPhinder in an attempt to predict host genera and species of these phage draft genomes and fragments. Table 5 provides an overview.

For six of the seven bacterial targets of the cocktail, HostPhinder predicted at least one phage targeting this type of bacteria. The only bacterium that was not predicted among the hosts was *Proteus*. Instead, the phage that was experimentally found to infect *Proteus* [38], was predicted as an *E. coli* phage with a coverage of 0.0026. This is not surprising, as the HostPhinder database contains no examples of *Proteus* phages. A *Sodalis glossinidius* was predicted, not corresponding to any of the anticipated targets. This bacterium is an endosymbiont of the tsetse fly [50] and its prediction was based on a coverage value of 0.43, where predictions with coverages above 0.2 have approximately 80% chance of being correct (see Figure 4 and Figure 8). The predicted hosts of the 4 phage fragments were generally based on a lower coverage than the 19 phage draft genomes, indicating that these predictions are less certain.

## 4. Discussion

In the present study, we developed a fast and simple method for prediction of phage hosts. Other studies have previously focused on the identification of phage-host pairs. Experimental methods examining phage-host interactions include mining viral signals from SAG (single amplified genomes) datasets; microfluidic digital PCR and phageFISH [55]. Recently, M. Martínez-García *et al.* combined single-cell genomics and microarrays technology to assign viruses to hosts depending on hybridization allowing for discovery of new virus-host pairs directly on a metagenomic samples without requiring cultivation or relying on genomic information [56]. In another study, Roux *et al.* developed a bioinformatics tool VirSorter [57], which was able to identify more than 12,000 virus-host linkages from publicly available bacterial and archeal genomes. In their study they analysed the virus-host adaptation in compositions in terms of mono- di- tri- tetra-nucleotide frequency and codon usage [58] showing the strongest signal of adaptation to host genome given by tetranucleotide frequency (TNF). A further classification method for phage host prediction, MGTAXA was developed by Williamson *et al.* in their metagenomic study of the marine microbe in the Indian Ocean [59]. MGTAXA links viral sequences to the highest scoring host taxonomic model based on polynucleotide genome composition similarity between phage and bacterial genomes. The software is not conveniently available anymore (as of December 2015) and we therefore could not compare its performance to HostPhinder’s. Finally, a recent publication by Edwards *et al.* reviewed the predictive power of several computational tools for predicting the host of a given phage based on genome information [60]. The authors highlighted the importance of such tools for the characterization of uncoltured virus from metagenomes, and found that homology-based approaches had the strongest signals for predicting phage-host interactions.

HostPhinder bases its predictions on co-occurring k-mers between the query phage genome and the genomes of reference phages with known hosts. Kmer-based approaches have recently been implemented for genome assembly [61], fast classification [62,63] and annotation [64] of metagenomes. Considering the highly mosaic structure of phage genomes, one of the advantages of using k-mers for phage host predictions is that the exact order of genetic elements does not influence the outcome, only their presence or absence.

On an independent evaluation set, HostPhinder was found to perform well, when predicting the hosts of phages currently found in public databases. A remarkable 74% accuracy for the host species and 81% for the host genus were obtained. Some hosts were consistently easier to predict than others. This was for example the case for *P. acnes*, where the host of all annotated *P. acnes* phages in the evaluation set were correctly predicted, while no non-*P. acnes* phages were erroneously predicted as such. The observation is in concordance with previous studies showing that *P. acnes* phages constitute a homogenous group, sharing 85% nucleotide sequence and having similar genome length [65,66]. Furthermore the examined *P. acnes* phages were not able to infect other members of the *Propionibacterium* genus [65,67]. For many of the mispredicted hosts of HostPhinder, the genus of the annotated and predicted host was the same, which might be considered concurrent with the ability of some phages to infect more than one species within a genus. Examples of such broad host range phages are *Salmonella* Phage Felix O1 [68], Mycobacteriophage D29 [69] and *Yersinia* Phage PY100 [70]. It is hence possible that the mispredicted phages are polyvalent, *i.e.*, capable of infecting more than one bacterial species. Alternatively they may represent actual misprediction by HostPhinder caused by closely related phages targeting different host species. In some cases, the host predicted by HostPhinder did not even belong to the same genus as the annotated host, e.g., the three *Yersinia* phages were all predicted to infect *Escherichia* with coverage values that indicate a reliable result, namely 0.57, 0.6 and 0.13. Indeed the genome sequence of the *Y. pestis* phage phiA1122 has been found to be closely related to coliphage T7, sharing 89% nucleotide identity [71]. Despite this high nucleotide identity, PhiA1122 is not able to infect *E. coli*, and has even been used by the Center for Disease Control and Prevention of the United States as a diagnostic agent to identify *Y. pestis* [72].

When applying HostPhinder to phage draft genomes and fragments from the INTESTI phage cocktail, the predicted hosts corresponded well with the advertised targets of the cocktail. One phage draft genome was, however, predicted to target *Sodalis glossinidius*, an endosymbiont of the tsetse fly. Excluding the remote possibility that phages targeting this bacterium has been added to the cocktail, it is likely that the HostPhinder prediction is incorrect or that the phage is able to infect *S. glossinidius* as well as one of the targets of the cocktail. A study by Ho-Won and Kyoung-Ho Kim has shown close relation in comparative genomic and phylogenetic analyses between EP23, a phage that infects *E. coli* and *Shigella sonnei* and, SO-1, which infects *S. glossinidius* [73]. It was, however, not examined if the phages were able to cross-infect the hosts.

Many phages have a very narrow host range and only target specific strains within a particular species. This feature has been used extensively previously, when typing, e.g., *S. enterica* [74] and *S. aureus* [75]. HostPhinder is not able to perform predictions beyond species level, partly due to the hosts of most phages in the public databases not being annotated beyond this. Further, to perform predictions down to specific strains of bacteria more factors than the mere genome resemblance would likely have to be taken into account, e.g., by examining the receptor binding proteins, identifying the number of restriction sites in the phage genomes or analysing the CRISPR regions of the host genome.

Another limitation to the performance of HostPhinder is the accuracy of the breadth of annotated host(s) of the references phages. Most of the reference phages had only one annotated host, although many examples exist of phages that are able to infect closely or even distantly related bacteria [76,77,78]. Further, the performance of HostPhinder depends on the size and completeness of the underlying database. As an example, at the time of compiling the database for this study, no *Proteus* phage genomes were available in public databases. Hence it is inherently impossible for the HostPhinder method to predict any query phage as a *Proteus* phage. Indeed, HostPhinder predicted an experimentally identified *Proteus* phage from the INTESTI phage cocktail as an *E. coli* phage, albeit based on a coverage value of 0.003 indicating that the prediction was not reliable. Carson *et al.* demonstrated the capability of a coli-proteus phage isolated from a Russian cocktail of equally eradicating *E. coli* and *Proteus mirabilis* biofilms [79], evincing the potential of some phages to infect both species. As more phage genomes become available, we will update HostPhinder database to ensure its continued high performance.

Despite the limitations in HostPhinder, we envision that the tool will be useful for narrowing down the list of potential hosts. With the growing availability of metagenome samples, new approaches are necessary to firstly identify phages and secondly, determine their host. Thanks to its capability of promptly identifying potential phage-host interactions, the HostPhinder tool has potential applications in ecology, human gut microbiocenosis studies, and other viral metagenomics analyses, where there is need to shed light on the nature of phages.

The current of HostPhinder is very simple, only taking into account genomic information about the phage. Further development of the tool will expand this, taking the genome of the host into account, which we expect will enable us to make predictions beyond host species level.

## 5. Conclusions

The current antibiotics resistance crisis warrants new ways to combat bacterial infections. For decades, phage therapy has been used for this purpose in countries belonging to the former Eastern Bloc, and to ensure transfer of the technology to the West, it is important to establish a pool of well-characterized phages. The presented HostPhinder method provides the phage community with an easy-to-use tool for predicting the host genus and species of query phages, usable when searching for phages with appropriate host specificity and for correlating phages and hosts in ecological and metagenomic studies. HostPhinder is freely available as a web server [1] and as a Docker image [2].

## Figures and Tables

**Table 1:** Overall performance of different similarity measures on ${\mathrm{phages}}_{\text{train-test}}$.

	Score	z	frac${}_{q}$	frac${}_{d}$	Coverage
Species (%)	77.03±0.112	77.81±0.111	77.24±0.111	78.43±0.111	78.76±0.108
Genus (%)	81.43±0.096	82.02±0.094	81.78±0.094	83.07±0.09	82.84±0.092

**Table 2:** Average and mean standard error of the overall HostPhinder performance over 100 ${\mathrm{phages}}_{\mathrm{train-test}}$ set resamplings with replacement.

Method	Criterion 1 (First Host)	Criterion 2 (Majority Host among Top-10)	Criterion 3 (Coverage Threshold, f=0.8)	Criterion 4 (Summing up Normalized Coverage Values, α=6.0)
Accuracy, Species (%)	78.76±0.108	74.79±0.102	79.1±0.104	79.13±0.105
Accuracy, Genus (%)	82.84±0.092	80.41±0.099	83.61±0.092	83.72±0.092

**Table 3:** List of host species (left) and genera (right), which HostPhinder predicts correctly.

Species	Representation in ${\mathrm{phages}}_{\mathrm{train-test,species}}$	Genus	Representation in ${\mathrm{phages}}_{\mathrm{train-test,genus}}$
*Enterococcus faecalis*	15	*Acinetobacter*	16
*Listeria monocytogenes*	21	*Listeria*	26
*Propionibacterium acnes*	21	*Propionibacterium*	24
*Vibrio cholerae*	35	*Streptococcus*	39
		*Streptomyces*	11
		*Thermus*	5

**Table 4:** HostPhinder and BLAST performance comparison on the ${\mathrm{phages}}_{\mathrm{eval}}$ set.

	BLAST	HostPhinder
No. of predictions, training on ${\mathrm{phages}}_{\mathrm{train-test},\mathrm{genus}}$	90%	97%
No. of predictions, training on ${\mathrm{phages}}_{\mathrm{train-test},\mathrm{species}}$	91%	96%
Accuracy on common predictions (GENERA) (%)	84.66 ± 0.188	85.13 ± 0.176
Accuracy on common predictions (SPECIES) (%)	76.92 ± 0.252	78.69 ± 0.237

**Table 5:** Overview of the results of HostPhinder predicting the hosts of 19 phage draft genomes (name starts with a “D” and *Proteus*) and 4 phage genome fragments (name starts with an “F”) from the INTESTI phage cocktail.

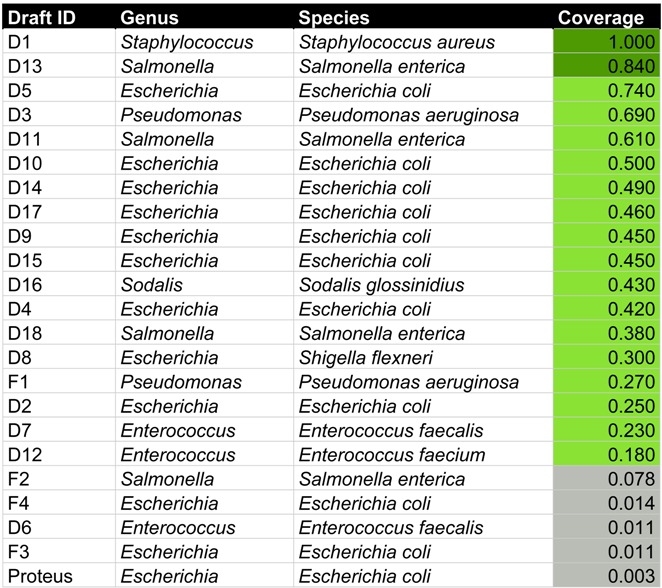

## Supplementary Files

Supplementary File 1
