# Supplementary material for: HostPhinder: A Phage Host Prediction Tool
Source: Viruses. 2016 May 4;8(5):116. doi: 10.3390/v8050116 (PMC4885074; doi:10.3390/v8050116)
Supplement: Supplementary File 1 [file viruses-08-00116-s001.pdf]

Supplementary Materials: HostPhinder: A Phage Host Prediction Tool

Julia Villarroel, Kortine Annina Kleinheinz, Vanessa Isabell Jurtz, Henrike Zschach, Ole Lund, Morten Nielsen and Mette Voldby Larsen

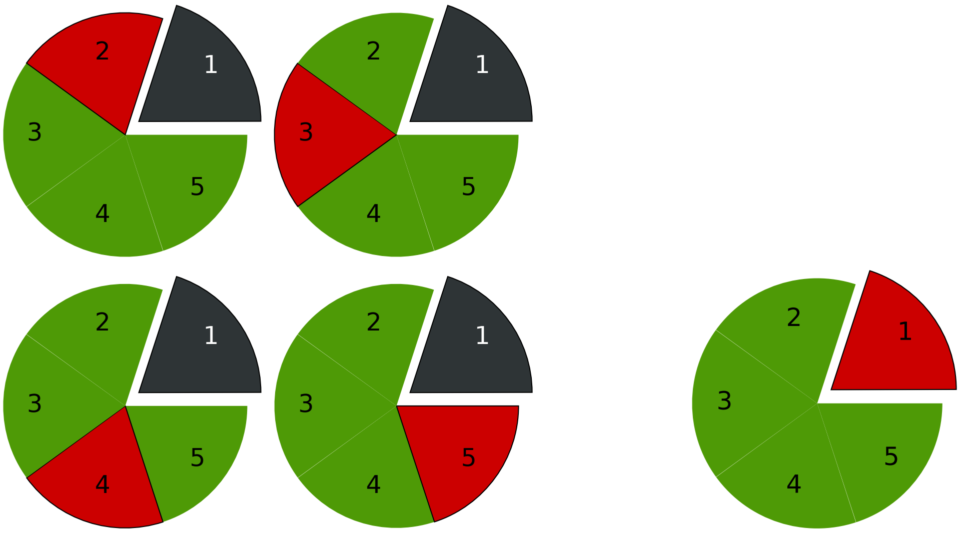

Figure S1. Four-fold cross-validation data partitioning. Left: group 1 in black, $phages_{eval}$, was left aside and alternatively 3 groups were used as training set, in green, to be tested on the remaining group in red. Green and red groups represent the $phages_{train\text{-}test}$ set. Right: Once the best similarity measure and criterion were determined, $phages_{eval}$ was predicted with HostPhinder trained on $phages_{train\text{-}test}$ for final evaluation.

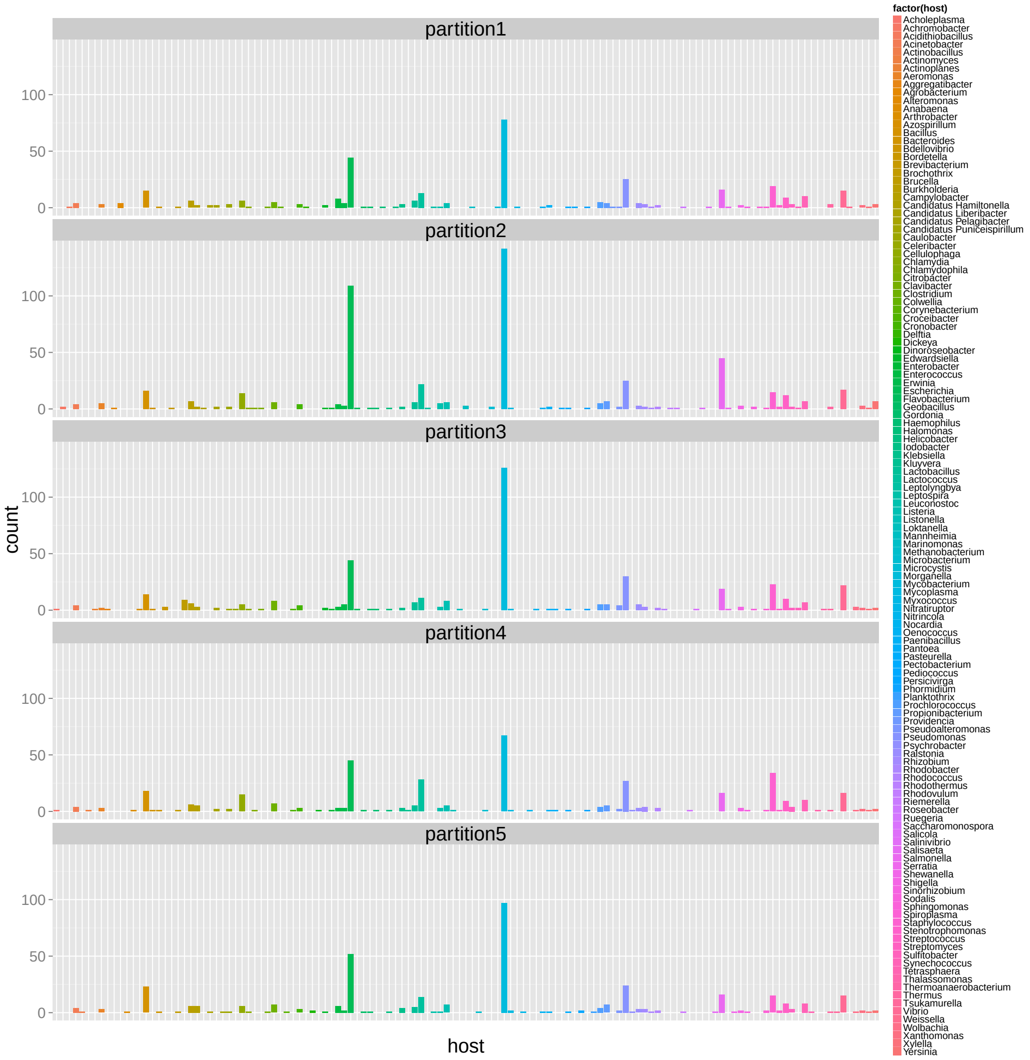

Figure S2. Host genera distribution between partitions.

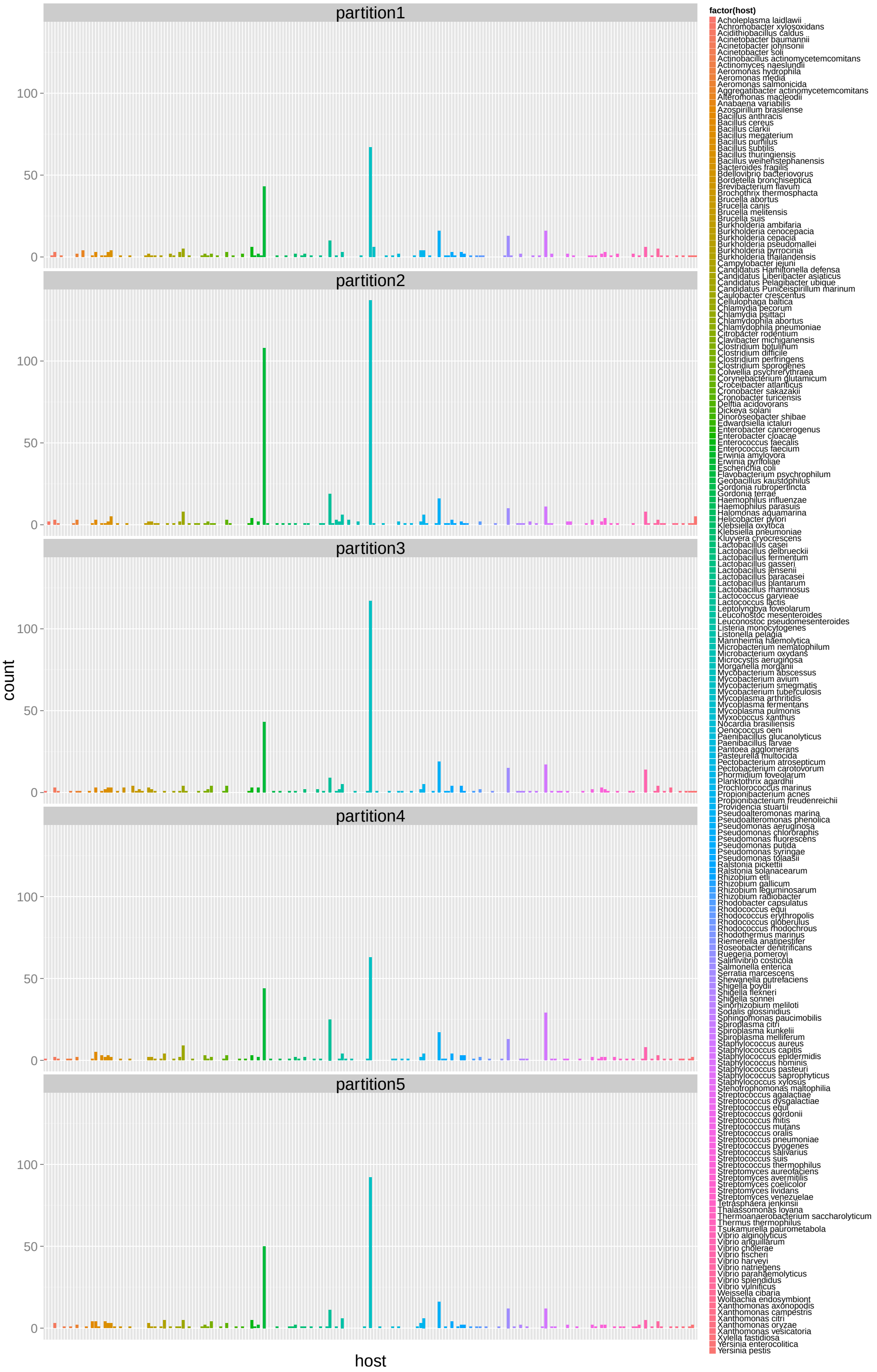

Figure S3. Host species distribution between partitions.

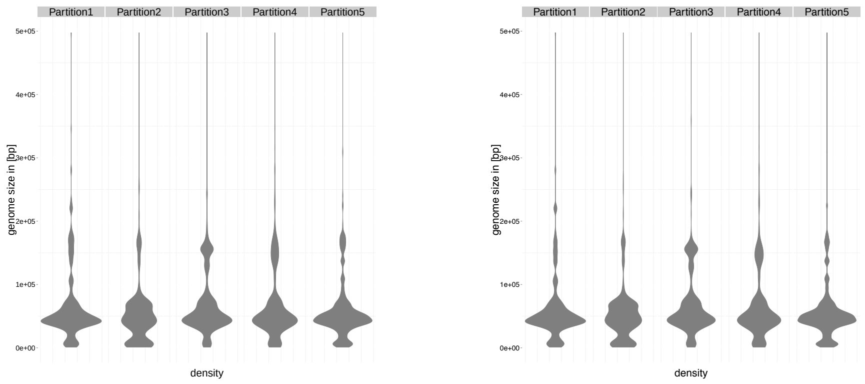

Figure S4. Genome size distribution between partitions in phages$_{\text{genus}}$ (**left**) and phages$_{\text{species}}$ (**right**).

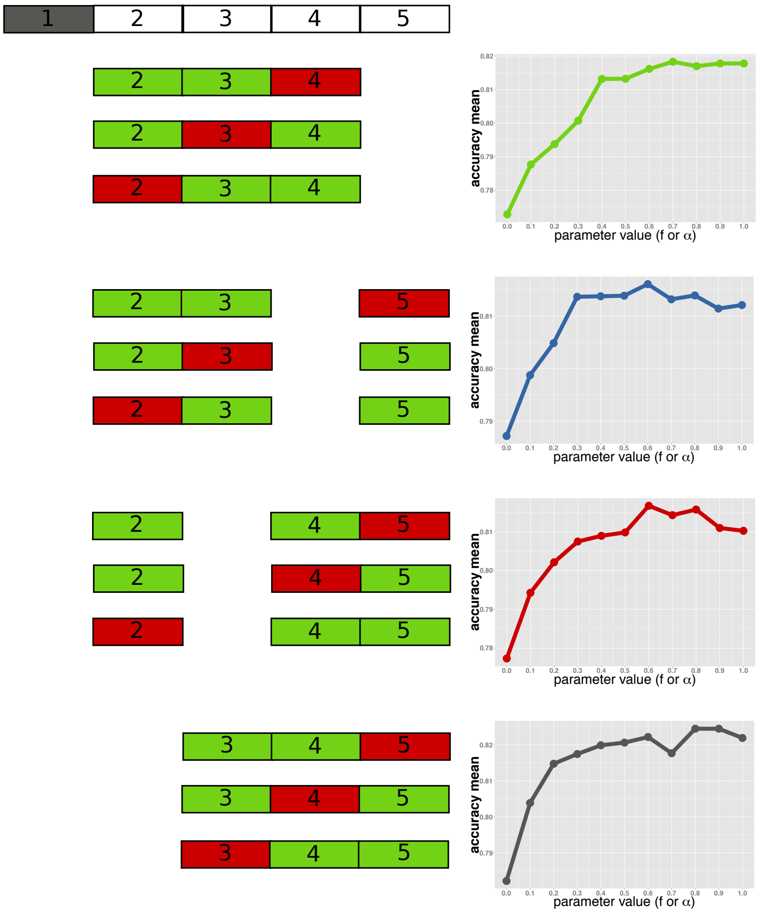

Figure S5. Three-fold nested cross-validation data partitioning. Among the 4 partitions in the phages$_{\text{train-test}}$ set (white boxes on the top), 3 were considered at a time for the inner cross-validation loop. Here in turn two partitions (green boxes) were used to train HostPhinder at different parameter values and to predict the third partition (red). The accuracy was calculated as right predictions over number of predicted queries in the 3 partitions used as test set. The accuracy average was calculated from 100 times bootstrap at different parameters values and plotted on an accuracy versus parameter value plot. Here the parameter is either f (criterion 3) or α (criterion 4). The same was repeated for the 4 tripartite cross validation loops.
